# Heat Shock Proteins: Potential Modulators and Candidate Biomarkers of Peripartum Cardiomyopathy

**DOI:** 10.3389/fcvm.2021.633013

**Published:** 2021-06-16

**Authors:** Graham Chakafana, Timothy F. Spracklen, Stephen Kamuli, Tawanda Zininga, Addmore Shonhai, Ntobeko A. B. Ntusi, Karen Sliwa

**Affiliations:** ^1^Department of Medicine, Faculty of Health Sciences, Cape Heart Institute, University of Cape Town, Cape Town, South Africa; ^2^Division of Cardiology, Department of Medicine, Faculty of Health Sciences, University of Cape Town, Cape Town, South Africa; ^3^Department of Biochemistry, Stellenbosch University, Stellenbosch, South Africa; ^4^Department of Biochemistry, University of Venda, Thohoyandou, South Africa; ^5^Cape Universities Body Imaging Centre, Faculty of Health Sciences, University of Cape Town, Cape Town, South Africa

**Keywords:** heat shock protein, stress, cardiomyopathy, pregnancy, biomarkers, drug targets

## Abstract

Peripartum cardiomyopathy (PPCM) is a potentially life-threatening condition in which heart failure and systolic dysfunction occur late in pregnancy or within months following delivery. To date, no reliable biomarkers or therapeutic interventions for the condition exist, thus necessitating an urgent need for identification of novel PPCM drug targets and candidate biomarkers. Leads for novel treatments and biomarkers are therefore being investigated worldwide. Pregnancy is generally accompanied by dramatic hemodynamic changes, including a reduced afterload and a 50% increase in cardiac output. These increased cardiac stresses during pregnancy potentially impair protein folding processes within the cardiac tissue. The accumulation of misfolded proteins results in increased toxicity and cardiac insults that trigger heart failure. Under stress conditions, molecular chaperones such as heat shock proteins (Hsps) play crucial roles in maintaining cellular proteostasis. Here, we critically assess the potential role of Hsps in PPCM. We further predict specific associations between the Hsp types Hsp70, Hsp90 and small Hsps with several proteins implicated in PPCM pathophysiology. Furthermore, we explore the possibility of select Hsps as novel candidate PPCM biomarkers and drug targets. A better understanding of how these Hsps modulate PPCM pathogenesis holds promise in improving treatment, prognosis and management of the condition, and possibly other forms of acute heart failure.

## Introduction

Protein folding processes are fundamental in the maintenance of cardiac tissue integrity (1). The metabolic and mechanical demands of the heart, such as its continuous contractile activities, place a burden for robust protein quality control systems (2). Several cardiovascular diseases such as ischemic heart disease and heart failure (HF) are characterised by increased mechanical and oxidative pressures which trigger an accumulation of misfolded proteins in cardiomyocytes. Misfolded proteins are toxic to cardiomyocytes, potentially causing cardiac insults that lead to HF (3). Under ensuing stress conditions, protein folding processes which typically occur in the sarcoplasmic reticulum, sarcomeres and mitochondria, are crucial in maintaining cardiac muscle integrity (4). Molecular chaperones such as heat shock proteins (Hsps) are an important class of proteins involved in the maintenance of proteostasis in various cell types under both normal and stress conditions. Here, we review the potential involvement of Hsps in peripartum cardiomyopathy (PPCM). Notably, the additional cardiac stress associated with pregnancy may further induce unique protein folding pathways in PPCM. Using bioinformatics tools, we further propose novel interactions (between proteins involved in PPCM pathogenesis and Hsps) which can be targeted toward drug interventions. Currently, echocardiography is the principal diagnostic tool for PPCM, as no reliable biomarkers exist. In this review, we also critically assess the potential of Hsps as candidate PPCM biomarkers.

## Pathophysiology and Molecular Pathways of PPCM

PPCM is a common and devastating disease that is associated with the unexpected loss of maternal cardiac function in the period surrounding parturition i.e., toward the end of pregnancy or within months following delivery (5). PPCM is characterised by a decreased left ventricular ejection fraction (LVEF) that is <45% in patients without prior cardiac disease (6). Generally, PPCM presents as congestive HF and systolic dysfunction, with typical symptoms including dyspnoea, fatigue, palpitations, oedema and chest pain (5). Although 90% of PPCM cases present after parturition, in rare cases disease onset has also been reported in the second trimester (7, 8). With a reported incidence ranging from 1:100 to 1:10,000 deliveries (9, 10), the frequency of PPCM appears to be influenced by ethnicity, with individuals of African origin at greater risk of developing the disease (10, 11). Other risk factors for PPCM include preeclampsia, multiparity, maternal age and multiple pregnancies.

Although the pathophysiology of PPCM is not entirely understood, several mechanisms of disease have been suggested. These include malnutrition, viral infection, autoimmunity and increased haemodynamic strain, although their roles in PPCM have proved nebulous (12). Current evidence, however, strongly suggests that PPCM may be driven by a pathological imbalance of pro- and anti-angiogenic hormones, as well as genetic factors. Two main pathways underlying disease pathogenesis have accrued from studies of mouse models of PPCM, as well as observations in human patients. These are reviewed in more detail elsewhere (12), but both involve the creation of a profoundly vasculotoxic environment through imbalances in angiogenic hormones.

The first pathway is characterised by increased expression of the pituitary hormone prolactin (PRL) which, in conditions of high oxidative stress, ultimately leads to cardiomyocyte apoptosis and cardiac dysfunction. The post translational processing of PRL is complex and the full length 23 kDa protein may be cleaved by peptidases (such as Cathepsin D) into a smaller 16 kDa variant. The 16 kDa PRL variant is a potent anti-angiogenic factor which acts as a vasoinhibin which can also cause vascular dropout, global systolic dysfunction and cardiac endothelial apoptosis. A murine PPCM model demonstrated that cardiac-specific deletion of the *STAT3* gene caused increased oxidative stress through reduced MnSOD expression (13). Further investigation also revealed an increase in cathepsin D activity and a corresponding increase in 16 kDa PRL levels (13). Altogether, this implies a vital role of STAT3 in cardioprotection during pregnancy, suggesting that dysregulation of STAT3 may also underlie PPCM. Recently, inhibition of Notch1/Hes1 has been found to induce PPCM through suppression of STAT3 activation, as well as increasing cathepsin D expression (14). Another protein involved in PPCM pathophysiology, Akt, is highly activated during pregnancy and promotes cardiac hypertrophy, and was shown to be activated by both PRL and interferon-γ (IFNγ) (15).

The second PPCM pathophysiology pathway involves the increased placental secretion of soluble Fms-like tyrosine kinase 1 (sFlt1) into the maternal system. Precisely why sFlt1 is secreted by the placenta is unclear, but both sFlt1 and membrane bound Flt1 are decoy receptors for vascular endothelial growth factors (VEGFs). VEGFA and VEGFB are proangiogenic factors and important mediators of cardiac homeostasis, but the binding of sFlt1 inhibits their activity (16). VEGF expression is driven by PGC-1α, and suppression of this in murine hearts led to PPCM and an increased susceptibility to sFlt1-induced cardiomyopathy (17). In this study, excessively high sFlt1 levels were able to cause cardiomyopathy, even in mice without the PGC1-1α deletion or pregnancy, indicating that excess sFlt1 alone can induce cardiac dysfunction. This emphasises the sensitivity of the heart to angiogenic imbalance as a result of placental sFlt1, that may occur during pregnancy. Synchtiotrophoblasts of the placenta secrete copious amounts of sFlt1 and as such, plasma levels of the protein rise exponentially toward birth (18). Most of the free VEGF in maternal circulation is thus neutralised by sFlt1 during pregnancy. More so, elevated sFlt1 have been described in women with PPCM (17), and have been directly correlated with disease severity and the occurrence of adverse clinical events (19). Notably, higher sFlt1 levels have been reported in twin pregnancies, another risk factor for PPCM (20, 21), possibly as a result of the larger placenta (22).

## Cardiac Mechanical Stress During Pregnancy

**Figure 1:**
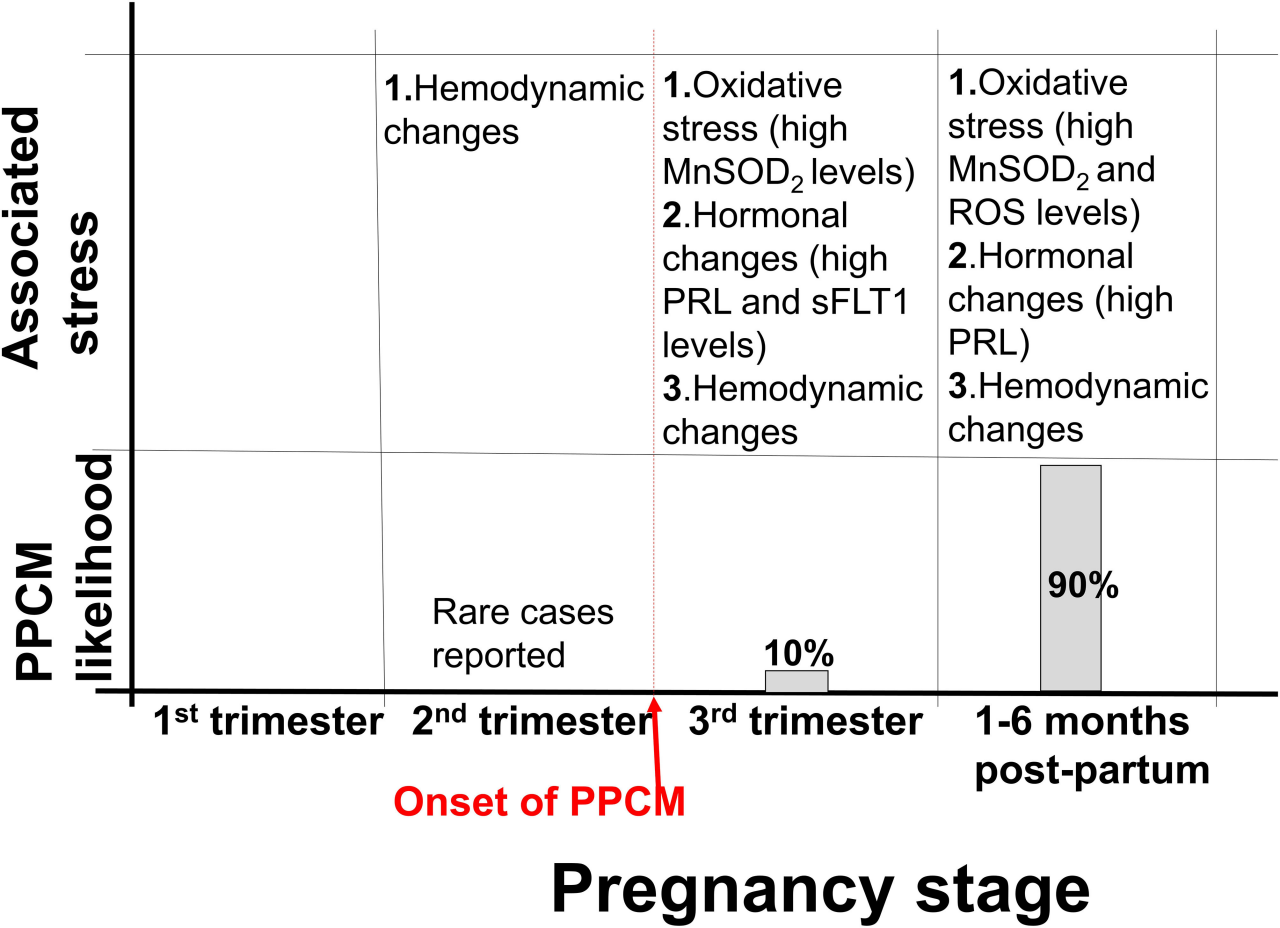
Stress associated with PPCM development. Approximately 10% of PPCM cases present within the third trimester, while 90% of PPCM cases present postpartum. Hemodynamic changes associated with pregnancy begin in the second trimester and persist to approximately 6 months postpartum. Several other stresses, such as hormonal changes are also associated with pregnancy.

Pregnancy is accompanied by dramatic hemodynamic changes, including reduced resistance during systole (afterload) and a 50% increase in cardiac output and blood volume (23). Furthermore, foetal microchimeric cells may reduce cardiac function resulting in increased cardiac mechanical stress during pregnancy (24, 25). It is however worth noting that most of these changes typically occur early in gestation, many months before PPCM typically presents (Figure 1). These changes trigger homeostatic and structural remodelling of cardiovascular tissues. Whereas, hemodynamic changes of pregnancy peak in the second trimester, hormonal changes of pregnancy are most drastic in the third trimester and early postpartum (Figure 1) (26). These changes also coincide with the presentation of PPCM. As such, the vasculotoxic hormonal changes that occur during and after parturition act as a trigger for PPCM. Indeed, several studies have demonstrated that PPCM is triggered by the rapidly changing environment of late gestation thus inducing vasculopathy in susceptible women (13, 27, 28). This is supported by the fact that hormones that likely trigger PPCM (PRL and sFlt1) are mostly at their peak in late pregnancy and postpartum. In addition, unlike other forms of cardiomyopathy, cardiac function is usually restored upon a drop in these hormones which comes with delivery. Apart from sFlt1, the placenta also secretes several other hormones which may result in maternal stress during pregnancy. Despite these cardiac demands associated with pregnancy, there is a need for protein quality control to be maintained in the cardiomyocytes.

## Cardiomyocyte Proteostasis and Potential Roles of Hsps in PPCM

**Figure 2:**
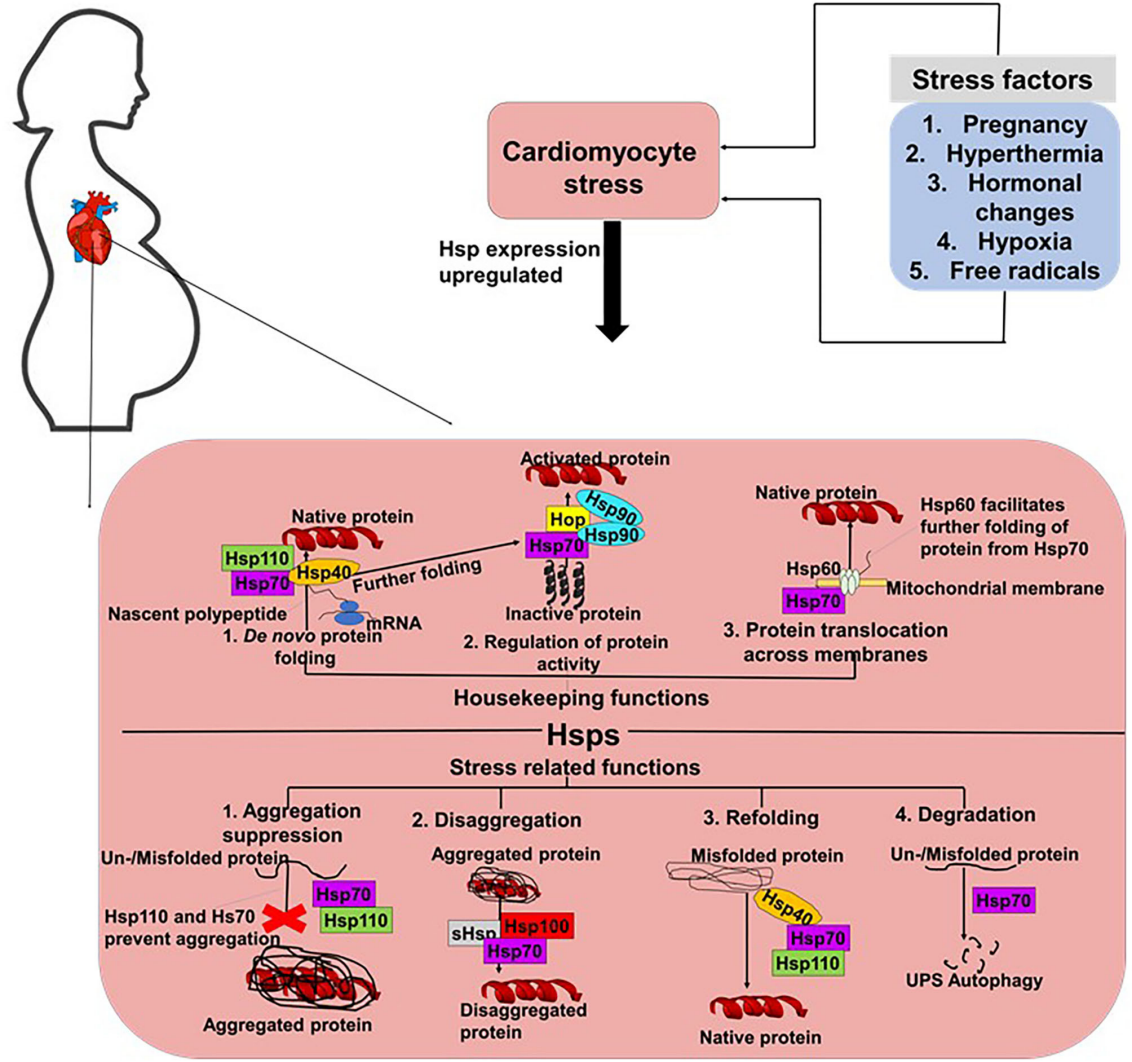
Proposed roles of Hsps in PPCM. In cells, Hsp70s perform both housekeeping and stress response related roles. Hsp70s co-operate with various co-chaperones such as Hsp40 and Hsp110 to facilitate the *de novo* folding of polypeptides from the ribosome to facilitate their folding into their native conformations. Hsp70s also co-operate with Hsp90 and Hop to activate proteins. Hsp70s also form partnerships with Hsp60 to facilitate the translocation of proteins across membranes. Under stress conditions, Hsp70s prevent the formation of aggregates and also facilitate the degradation of misfolded/unfolded proteins.

The robust maintenance of proteostasis in cardiomyocytes is crucial in ensuring the integrity of cardiac tissue. An accumulation of misfolded and unfolded proteins results in the formation of aggregates which are usually cytotoxic. The heart is constantly exposed to mechanical stresses associated with its continuous contractile activities, as well as chemical stresses induced by free radicals and hormones. In PPCM, pregnancy further burdens cardiac tissue, as the heart readjust to the needs of the developing foetus. Stress supresses the cell's capacity to maintain proteostasis thus compromising the ability of proteins to attain native conformation. Cellular stress can lead to protein misfolding or unfolding, leading to proteins that are unable to carry out their normal functions (29). Such stresses typically impair protein folding, potentially resulting in the formation of functionally impaired and toxic protein aggregates that trigger cardiac insults. Under the ensuing stress conditions, molecular chaperones such as Hsps likely facilitate cardiomyocyte proteostasis. Generally, Hsps perform a myriad of housekeeping and stress-protective roles in cells to maintain proteostasis (Figure 2) (30). We propose that Hsps are particularly important in PPCM since the heart is further burdened by pregnancy related stresses that may impair optimal protein folding processes in the cardiomyocytes.

Hsps can generally be classified into seven families, based on structural and functional features (Table 1). Broadly, Hsps function to facilitate the correct folding and assembly of polypeptides, thus preventing the formation of misfolded or incorrectly assembled proteins (44). Hsps also play an important role in the suppression or inhibition of polypeptide aggregation in cells (45). When a nascent polypeptide chain exits the ribosome or an organellar import pore, or when a labile native protein becomes transiently heat denatured, it may transiently unfold and expose hydrophobic segments to the aqueous environment (31). Depending on the intensity and duration of the stress as well as the degree of hydrophobic exposure, the misfolded monomers may clamp together through intermolecular hydrophobic associations to form aggregates (46). Hsps of the “holdase” class (such as small Hsps (sHsps), Hsp40, Hsp70, and Hsp110) can bind to the exposed hydrophobic residues on the surface of misfolded polypeptides to prevent the formation of aggregates (47).

**Table 1:** Major Hsp families and cardiovascular roles.

**Protein family**	**Localisation**	**CVD implication**	**Stress inducers**	**References**
Hsp110	Cytosol, ER	ND^*^ (General role: Protein aggregation suppression; Possess holdase function)		(31)
Hsp100	Mitochondrion	ND^*^ (General role: Dis-assembly of quaternary structure of polypeptide complexes and are required for thermotolerance)		(32)
Hsp90	Myocyte	1. Hsp90 antibody levels rise 16-fold under stress (potential CVD biomarker?) 2. Hsp90 supports Akt signalling (elevated Hsp90 and Akt levels have been reported in hypoxia challenged cardiomyocytes) 3. Hsp90 possesses anti-apoptotic effects on hypoxia-mediated cardiomyocyte damage 4. Cardiac Hsp90 supports protein maturation and has roles in the development of mutation-related cardiac arrhythmia 5. Hsp90 regulates angiotensin II-induced cardiac hypertrophy	Ischemia ROS	(33, 34)
Hsp70	Myocyte cells	1. Specific role in myocardial protection from chronic ischemia 2. Participates in myocardial adaptive processes to chronic repetitive ischemia (high tissue levels of Hsp72 have been reported in myocardial hibernation) 3. High Hsp70 expression levels were correlated to HF progression (potential HF biomarker)	Ischemia and mechanical stress, steroid hormones, drugs, physical exercise	(35, 36)
Hsp60	Endothelial cells Myocyte surface	1. Ischemic myocardial damage2. Thought to participate in inflammatory processes (activates autoimmune response. High Hsp60 expression elicits an autoimmune response that can trigger further vascular/ myocardial damage) 3. High Hsp60 levels reported in coronary artery disease patients 4. Serum Hsp60 was related to the severity of CHF and associated with a high risk for late stage cardiac events in CHF patients	Biochemical or infective insults; hyperthermia	(37–40)
Hsp40	Cytosol, membranes, ER	1. Co-chaperone of Hsp70; Host cell modifications 2. Associated with development of fatal DCM	Ischemia and mechanical stress	(41)
sHsps		1. High expression levels during cardiac hypertrophy 2. Cardioprotection (Hsp27 has a cardioprotective effect in cases of infarction) 3. Overexpression of Hsp27 results in reduction in cell apoptosis in cardiac tissue 4. High Hsp25 expression improved survival of cardiomyopathy patients and the heart resistance against toxicity	Hypertrophic stimuli, including aortic banding, angiotensin II infusion	(42, 43)

* *ND, not determined*.

The roles of Hsps in several cardiovascular conditions such as HF and dilated cardiomyopathy (DCM) have been reported (Table 1). The Hsp70 family of molecular chaperones is a central hub for the maintenance of proteostasis in cells (48). Hsp70s are actively involved in almost every stage of a protein's life course (Figure 2). Thus, Hsp70s facilitate folding of nascent peptides emerging at the ribosomes (49), protein trafficking and translocation across membranes (50). In addition, Hsp70s facilitate the refolding of misfolded protein (31, 51), and also channelling misfolded proteins which are beyond repair toward degradation (52, 53). In order for Hsp70s to function efficiently, they depend on assistance from functional networks formed with members of several co-chaperones which include Carboxyl terminus of HSC70-interacting protein (CHIP), Bcl-2 associated athanogene 3 (BAG3) and Hsp70-Hsp90 organising protein (Hop) (2). Additionally, Hsp70 also forms functional networks with Hsp40 (54), Hsp90 (55), and sHsps (56). Protein folding by Hsp70 is tightly controlled by J-domain proteins (Hsp40) and nucleotide exchange factors (NEFs) such as BAG3. The co-ordinated action of Hsp70 and Hsp90 facilitates the folding of most structural and signalling proteins (Figure 2). In fact, Hsp70 has been described as a promiscuous chaperone that is capable of binding virtually any peptide sequence (57).

**Figure 3:**
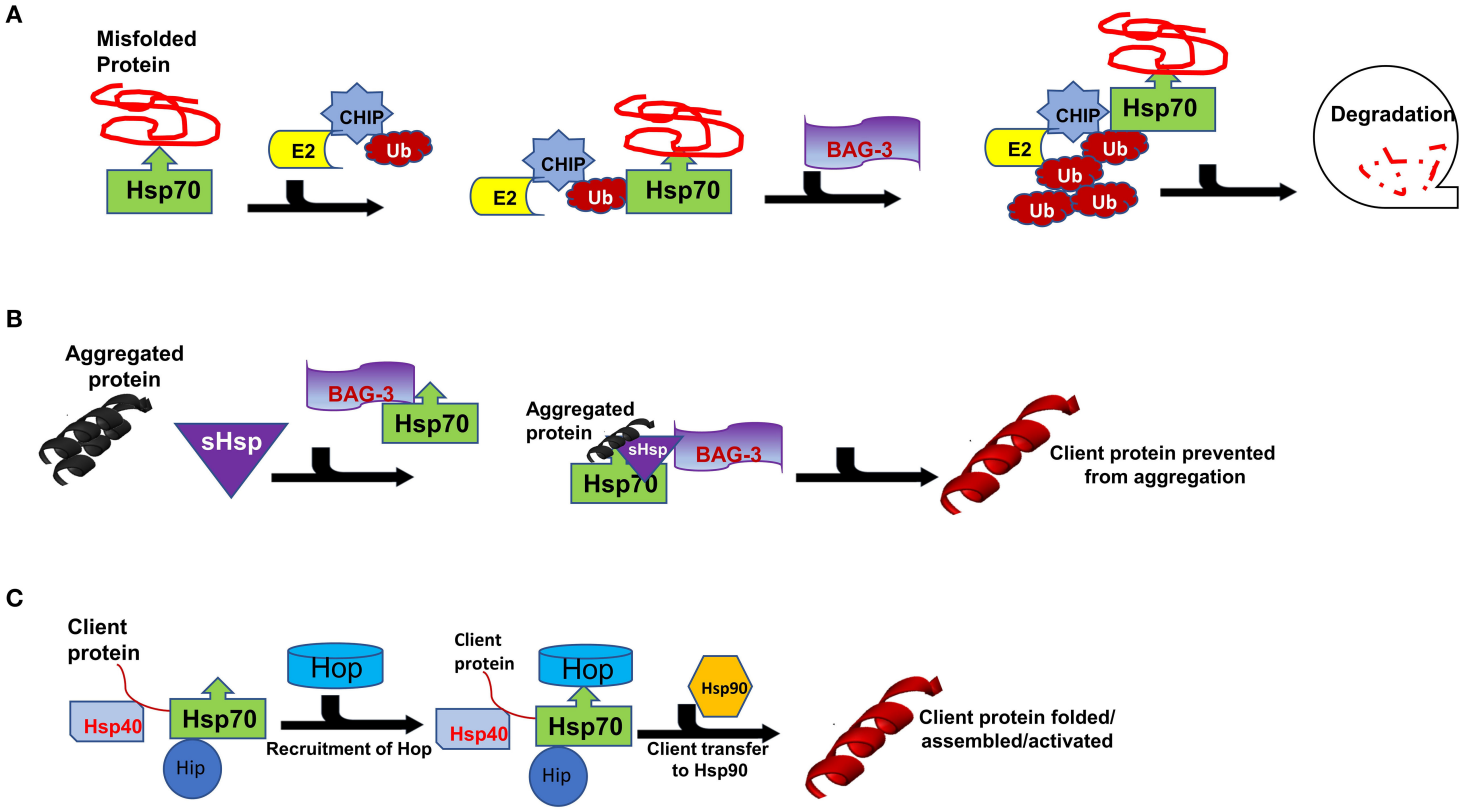
Interaction of Carboxyl terminus of HSC70-interacting protein (CHIP) with binding partners for protein quality control. Hsp70 functions with co-chaperones CHIP, BAG3 and Hop in maintain cellular proteostasis. **(A)** The CHIP pathway involves the association of CHIP with Ub, E2, BAG3, Bcl-2-associated athanogene 3 and Hsp70 to facilitate degradation. **(B)** Formation of sHSPs-BAG3-HSP70 complex to prevent aggregation of proteins thus forming insoluble substrates in the cardiomyocytes. **(C)** Hsp70-Hsp90 Organising Protein (Hop) adaptor properties to link Heat shock protein 70 (Hsp70) with Heat shock protein 90 for client substrates transfer.

Hsps also play crucial roles in the ubiquitin proteasome system (UPS) which is the main proteolytic system in eukaryotic cells facilitating the degradation of misfolded proteins. It has previously been established that the constitutively expressed Hsp70 (Hsc70) is required for ubiquitylation of several proteasome substrates (58). Furthermore, the majority of E3 ligase complexes of the UPS pathway have been shown to cooperate with Hsps (59). Hsps also function as escort factors that either deliver or dock the Ub-protein conjugates to the proteasome thus preventing the formation of ubiquitylated protein aggregates (Figure 3A) (53). Functional co-operations of Hsp70 could be pivotal in maintaining cardiomyocyte proteostasis during pregnancy-induced stress. The co-chaperone CHIP (a ubiquitin ligase) is ubiquitously expressed, although it is prominently expressed in striated muscle such as cardiac tissue (2). Functionally, CHIP co-operates with Hsp70 to ubiquitinate misfolded proteins that cannot be repaired, targeting them for protein degradation by the proteasome (Figure 3A) (60). Previous studies have implicated CHIP in cardiac disease. In a murine model, genetic knockout of CHIP resulted in exaggerated cardiac hypertrophy, as evidenced by increased heart weights, wall thickness and cardiomyocyte size following exercise or pressure overload (61, 62). Genetic knockout of CHIP was also associated with a dramatic decline in cardiac function in response to pressure overload (63). Due to its ability to regulate Hsp70 chaperone activity, CHIP may also be an important determinant in modulating Hsp70-chaperoned proteins in the cardiomyocyte.

The interaction of Hsp70 with another co-chaperone, BAG3, is also critical for cardiac muscle development and vascular disease pathogenesis (64). BAG3 is generally involved in a range of cellular functions which include protein folding, apoptosis, autophagy as well as CMA/chaperone-assisted selective autophagy (CASA) to the UPS (2). BAG3 not only forms functional co-operations with Hsp70, but also with sHsps such as HspB5, HspB6 and HspB8 (65, 66). sHSPs act in concert with Hsp70 to facilitate protein refolding (Figure 3B) (67, 68). Since sHSPS lack ATP-dependent enzymatic activity that is necessary for active protein refolding, BAG3 facilitates the formation of a BAG3-sHsp-Hsp70 complex through which protein refolding can occur. The direct involvement of BAG3 in cardiac disease has previously been reported in ΔBAG3 mice that were observed to develop cardiomyopathy and non-inflammatory myofibrillar myopathy (MFM) (64).

In the heart, Hsp70 is induced by several factors which include steroid hormones (e.g., vasopressin), free radicals, drugs, probiotic derived proteins, physical exercise and environmental changes (69, 70). Elevated Hsp70 expression results in reduced myocardial infarction (71). In addition, Hsp70 levels are also thought to correlate with a timecourse of cardioprotection (36). Furthermore, high Hsp70 levels are linked with a decrease in cardiac apoptosis (33, 72). As such, high Hsp70 levels have been reported to confer improvement in the recovery of post ischemia reperfusion injury (73). Wei et al. (74) demonstrated high Hsp70 expression levels in hearts showing failure due to cardiomyopathy (arrhythmogenic cardiomyopathy, DCM) and ischemia. The degradation of misfolded proteins is important for the constant turnover of sarcomeric proteins required for correct function and regulation of cardiac mass (75). In addition, Hsp70 co-operates with CHIP and BAG1 to control the degradation of myosin and sarcomeric proteins (76). Given these roles of Hsp70 in cardiac function, it is tempting to speculate implications of the chaperone in PPCM. This hypothesis may need experimental validation.

Hsp90 is a unique molecular chaperone which possesses the ability to bind target proteins that are in a near native state in order to mediate the final stages of folding (77). In addition, Hsp90 is specialised to facilitate the folding of specific, defined sets of client proteins such as steroid hormone receptors, TLR innate immunity receptors, RNA polymerases and PI3-kinase-related kinases (PIKKs) (78). As such, Hsp90 modulates cell signalling, genome maintenance and assembly of transcriptional and translational apparatuses in cells (36). Together with Hsp70, Hsp90 plays key roles in protein folding as they facilitate the folding, maturation and activation of virtually all proteins in the cell (50, 57). While Hsp70 generally binds to nascent polypeptide chains at the ribosome, Hsp90 binds a more specialised clientome that includes steroid hormones and kinases. Hop acts as an adaptor protein to enable Hsp70-Hsp90 interaction through their C-terminal sequences (Figure 3C) (79, 80). As such, Hop enables the transfer of client proteins such as kinases, nuclear receptors like the steroid hormone receptors (SHR) and transcription factors between the chaperones Hsp70 and Hsp90 for folding, assembly and activation (Figure 2) (81, 82).

Hsp90 also plays an active role in protein quality control by directing misfolded proteins toward the UPS for degradation (83). Hsp90 possesses anti-apoptotic effects on hypoxia-mediated cardiomyocyte damage (84). An Hsp90 client, ErBB2, was shown to be linked to the development of HF in a murine model (85). Mice lacking cardiac specific ErBB2 developed HF characterised by left ventricular (LV) dilation, wall thinning and decreased systolic function (85). Due to its large clientome, we speculate a likelihood of Hsp90 in chaperoning several other proteins involved in PPCM pathogenesis. We therefore sought to predict the interaction and roles of Hsp90, Hsp70 and sHsps in PPCM using bioinformatics.

## Predicted Associations Between Major Hsp Families and the PPCM Proteome

**Figure 4:**
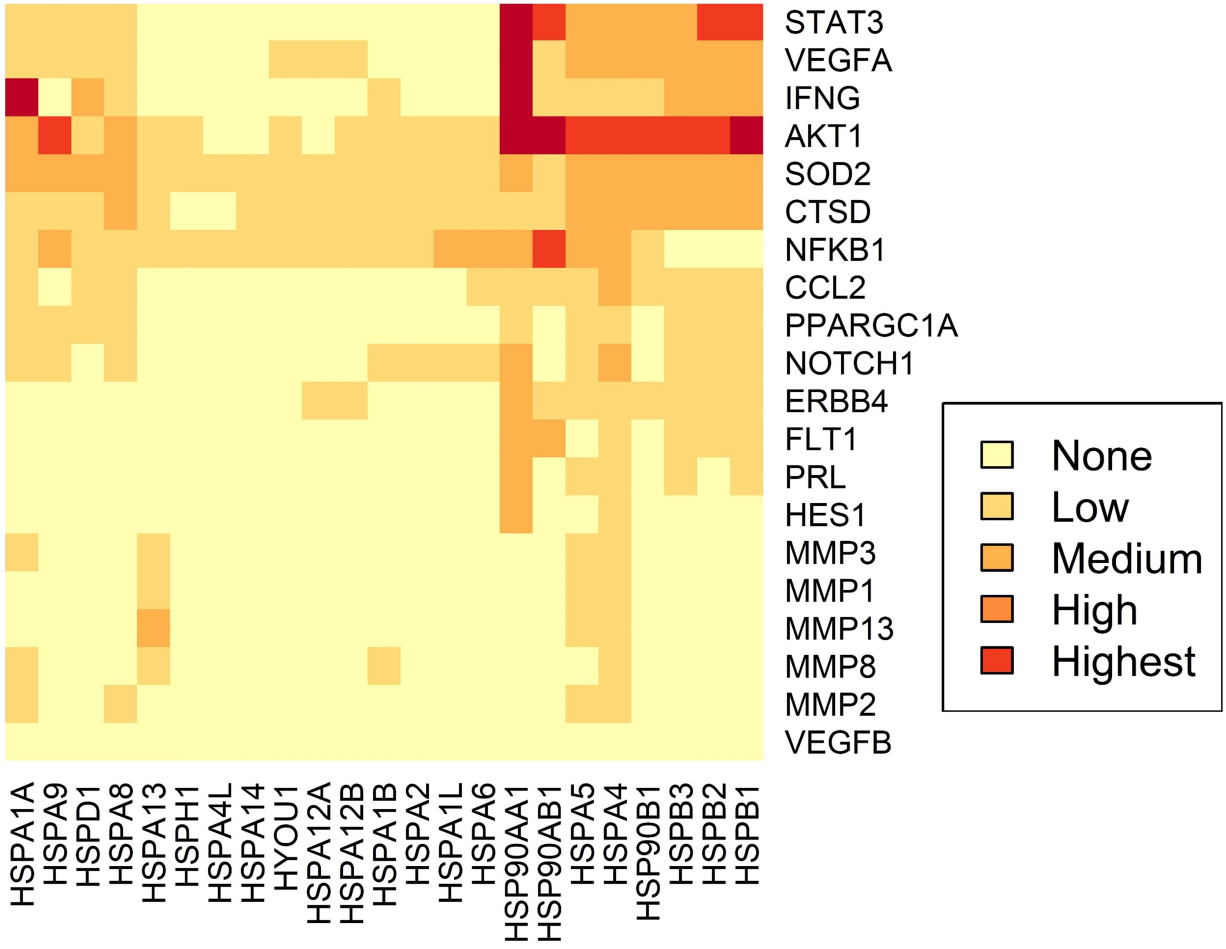
Predicted interactome of several Hsps with PPCM proteins. The heat map shows the interaction of PPCM pathogenesis proteins with Hsp70, Hsp90 and sHsp group members.

Using STRING analysis, we predicted the associations of several proteins currently known to be involved in PPCM pathophysiology with members of the Hsp70, Hsp90 and sHsp family members (Table 2 and Figure 4). Although the actual implications of such interactions are at present unknown, experimental validation may provide insights into candidate drug design or biomarker discovery. These Hsps and PPCM protein associations are described in the following sections.

**Table 2:** STRING interactions between heat shock and PPCM proteins.

**Protein**	**Hsps involved**	**Role in PPCM pathology**
Akt	HspA4, HspA5, HspA9, Hsp90, sHsps	Accelerates inflammation and fibrosis postpartum, through an unknown mechanism
CCL2	HspA4	Initiates inflammatory process, triggered by IFNγ or PRL
ERBB4	Hsp90	Cardiomyocyte survival; suppression during PPCM triggers cellular apoptosis
Flt1	Hsp90	Excess of soluble form (sFlt1) triggers angiogenic imbalance and PPCM, associated with adverse outcomes in PPCM patients
HES1	HspA4, Hsp90	Activation of cardioprotective STAT3 signalling
IFNγ	HspA1A, Hsp90	Continuously high IFNγ serum levels are associated with increased inflammatory status and adverse outcomes in PPCM patients
MMP	HspA13	MMPs can cleave PRL to its 16 kDa variant. High MMP levels have been detected in murine PPCM experiments
MnSOD	HspA4, HspA5	Responsible for organ specific antioxidant defence mechanisms in the peripartum phase. Generally cardiac MnSOD levels are high, although these are reduced in PPCM patients
NF-κB	HspA1L, Hsp90	Transcription factor activated by 16 kDa PRL, inducing apoptosis and antiangiogenic effects
NOTCH1	Hsp90	Activation of cardioprotective STAT3 signalling
STAT3	Hsp90, sHsps	Cardioprotective signaling
VEGF	HspA4	Crucial for blood vessel formation and homeostasis (VEGF A). Also drives trans-endothelial transport of fatty acids in the cardiomyocytes (VEGF B)

### Akt

Akt is a protein kinase and a key component of the ubiquitous PI3K/Akt signalling pathway. Activated Akt promotes cellular survival, proliferation and growth. In the heart, Akt signalling induces both pathological hypertrophy and physiological hypertrophy (for example, during pregnancy) (86). However, Akt appears to have an exacerbatory effect in PPCM (15), possibly by aggravating an underlying angiogenic imbalance. Select Hsp70 (HspA4, HspA5, HspA9), Hsp90 (Hsp90AB1), and sHsps (HspB1) are predicted to interact with Akt (Table 2).

HspA4 (a non-canonical Hsp70 member), HspA5 (an Hsp of the endoplasmic reticulum (ER) also referred to as Grp78), and HspA9 (a mitochondrial Hsp also referred to as Grp75) (Figure 4) have been demonstrated to activate Akt in cancer models (87–90). Interestingly, Akt has also been shown to phosphorylate HspA5 (91) and regulate its expression (92), thus suggesting a possible feedback loop in which Akt and Hsp70 proteins positively regulate each other. This may be protective in cellular conditions such as oxidative stress and ER stress, which induce HspA9 and HspA5, respectively (89, 90). Since these Hsp70 isoforms are predicted to bind and possibly activate Akt, it is possible that HspA4, HspA5, and HspA9 play decisive roles in PPCM pathogenesis. HspA5/Akt interaction has been demonstrated as essential for cardiac development, function, and stress response. The loss of HspA5 in cardiomyocytes leaves them vulnerable to apoptosis following oxidative and ER stresses through suppressed Akt signalling (93), while upregulation of HspA5 attenuated ischaemia/reperfusion-induced cardiac damage by stimulating Akt activity (94, 95). Recently, ER stressors and ischaemia were also shown to increase the secretion of HspA5 by cardiomyocytes (96), indicating an extracellular cardioprotective role of this Hsp by activating Akt signalling.

Hsp90 proteins and sHsps are also predicted to interact with Akt (Figure 4). Similar to Hsp70s, these also appear to be involved in the stabilisation and activation of Akt kinase activity (97–100). Hsp90AB1/HspB1 interaction with Akt may have cardioprotective effects against a range of cardiac insults (101–105). While the activation of Akt by Hsps is generally beneficial in response to several cardiac insults, Akt stimulation is detrimental in the case of PPCM (15). As PPCM is characterised by oxidative and other stresses (Figure 2), the induction of Hsp70s, Hsp90s and sHsps during PPCM is highly probable, as is the subsequent stimulation of the Akt signalling pathway, although more research will be needed to confirm this hypothesis.

### STAT3

Contrary to Akt, STAT3 activation may inhibit PPCM (13). Although no interactions between Hsp70s and STAT3 were observed, members of the Hsp90 and sHsp families are predicted to interact with STAT3 (Figure 4). The role of Hsp90 and the small HspB1 (Hsp27) in STAT3 signalling has recently been reviewed in detail (106), with these Hsps acting as key chaperones at numerous stages of the STAT3 pathway, including phosphorylation, activation and nuclear localisation of STAT3, as well as limiting its proteasomal degradation. It is therefore plausible that Hsp90 promotes STAT3 activation which may reverse the progression to fulminant PPCM. Therapeutic interventions that promote elevated Hsp90 expression levels in the cardiomyocytes may therefore prove beneficiual to PPCM patients. Further research is however needed to ascertain the functions of Hsps in modulating these pathways.

### Other Interactions

Several other interactions were observed between Hsps and proteins in the PPCM pathway (Table 2), although the functional and biochemical roles of these remain to be determined. For instance, excess sFlt1 and reduced VEGF levels are key components of the angiogenic imbalance that characterises PPCM. While an interaction between Hsp90 and Flt1 was observed, current knowledge has only implicated Hsp90 in the maintenance of membrane-bound Flt1 in endothelial cells (107); interaction with the soluble form of the protein is unknown. However, because inhibition of Hsp90 could reduce levels of Flt1 (107), the role of Hsps in the stabilisation or folding of Flt1 may be worth exploring, especially in the context of placentally-derived sFlt1. We also predicted association of HspA4 with VEGFA. It has previously been demonstrated that HspA4 has a stabilising effect on *VEGFA* mRNA in cancer cells (108). It is conceivable that HspA4 suppresses aggregation of VEGFA under stress conditions, keeping the protein in a folding competent form, as has been demonstrated with other Hsp70 isoforms (56).

### The Role of Hsps in Buffering Mutations

PPCM has been demonstrated to have a genetic basis, in at least a subset of patients (109–111). It is notable that molecular chaperones play a key role in the translation of genetic variation, by silencing or potentiating mutations (112). Hsp90, in particular, may potentiate mutations by assisting the folding and function of the mutant proteins, allowing them to have immediate phenotypic consequences (113). Alternatively, Hsp90 can silence mutations so that they have no phenotypic manifestation, although these buffering effects can be overwhelmed by environmental stresses (113). The implications of this are that Hsp90, or other Hsps, can buffer genetic mutations in a manner that is dependent on the environmental conditions. This may explain, in part, why the same mutations can cause DCM in some individuals, and PPCM in others.

The modulation of mutations by Hsps has also been described in Fanconi Anaemia, where Hsp70 was found to bind to inactive mutant proteins with severe phenotypic effects (114). The roles of Hsps in the buffering of cardiomyopathy-causing mutations is largely unknown, although heritable cardiomyopathies including DCM and hypertrophic cardiomyopathy (HCM) may be characterised by dysfunction of the UPS and other protein quality control mechanisms (115, 116). DCM- and HCM-causing mutations typically occur as truncations of genes encoding the sarcomeric proteins titin and cMyBP-C, respectively, and mutations in both have been described in PPCM patients, as well as other sarcomeric gene mutations (109–111, 117). Notably, these truncated protein products are not incorporated into the sarcomeres of mutation carriers (118, 119), although in the case of titin this haploinsufficiency has been attributed to mRNA degradation by nonsense-mediated decay (120). The role of Hsps in the buffering of truncating PPCM-causing mutations remains to be determined.

### The Role of Hsps in the PPCM Inflammasome

In addition to their proteostatic roles, Hsps may also act as “chaperokines” which present antigens to the immune system. Hsp70, Hsp90 and sHps are secreted under stress conditions, where they can have pro- or anti-inflammatory effects [reviewed in (121)]. As myocardial inflammation is thought to be a key contributor to PPCM pathogenesis (12), the immunomodulatory effects of Hsps may be of interest. Indeed, several studies have demonstrated potential roles of Hsps in cardiac inflammatory pathways in response to myocarditis (122, 123) and myocardial injury (discussed below).

Extracellular Hsp70 can induce cardiomyocyte inflammation and cell death, in contrast with the pro-survival role of intracellular Hsp70 (124). Increased levels of circulating Hsp70 have been reported in models of acute myocardial infarction, autoimmune myocarditis and left ventricular dysfunction, all in association with elevated inflammatory markers and worse outcomes (37, 125, 126). Up-regulation of Hsp70 by treatment with Melusin in a mouse model of myocardial infarction was shown to reduce inflammatory cell infiltrates in the myocardium and improve cardiac function (127). The roles of Hsp90, Hsp60 and sHsps in cardiac inflammatory responses has also been demonstrated. Hsp90 has been shown to have cardioprotective effects in ischaemic pre- and post-conditioning by suppression of immune responses (128, 129). On the other hand, HspB1 may down-regulate leukocyte recruitment and cardiac inflammation (130). Hsp60 appears to induce cardiac inflammation and cytokine production (131, 132). It is therefore plausible that the level of circulating Hsps may promote or suppress cardiac inflammation, although it is unclear at this stage whether Hsp induction in PPCM would be beneficial or detrimental to recovery of cardiac function.

## Targeting Hsps Toward Novel PPCM Therapy

To date, no disease-specific interventions for PPCM exist. As is the case with other cardiomyopathies, PPCM management is primarily focused on managing volume status, neutralising neurohormonal maladaptive responses, and preventing complications (133). One of the most used PPCM treatments is bromocriptine which functions by suppressing PRL production postpartum. A small proof-of-concept randomised trial of bromocriptine in 20 women with PPCM in Africa demonstrated improvements in mortality and LVEF at 6 months (134). Those observations were confirmed in a multi-center randomized study (135). Based on these data the 2018 ESC Guidelines for the management of cardiovascular diseases during pregnancy states that bromocriptine may be considered in women with newly diagnosed PPCM (5). However, more research in this area is needed. As such, there is an urgent need for the identification of novel PPCM drug targets in the design of novel therapeutic interventions.

**Figure 5:**
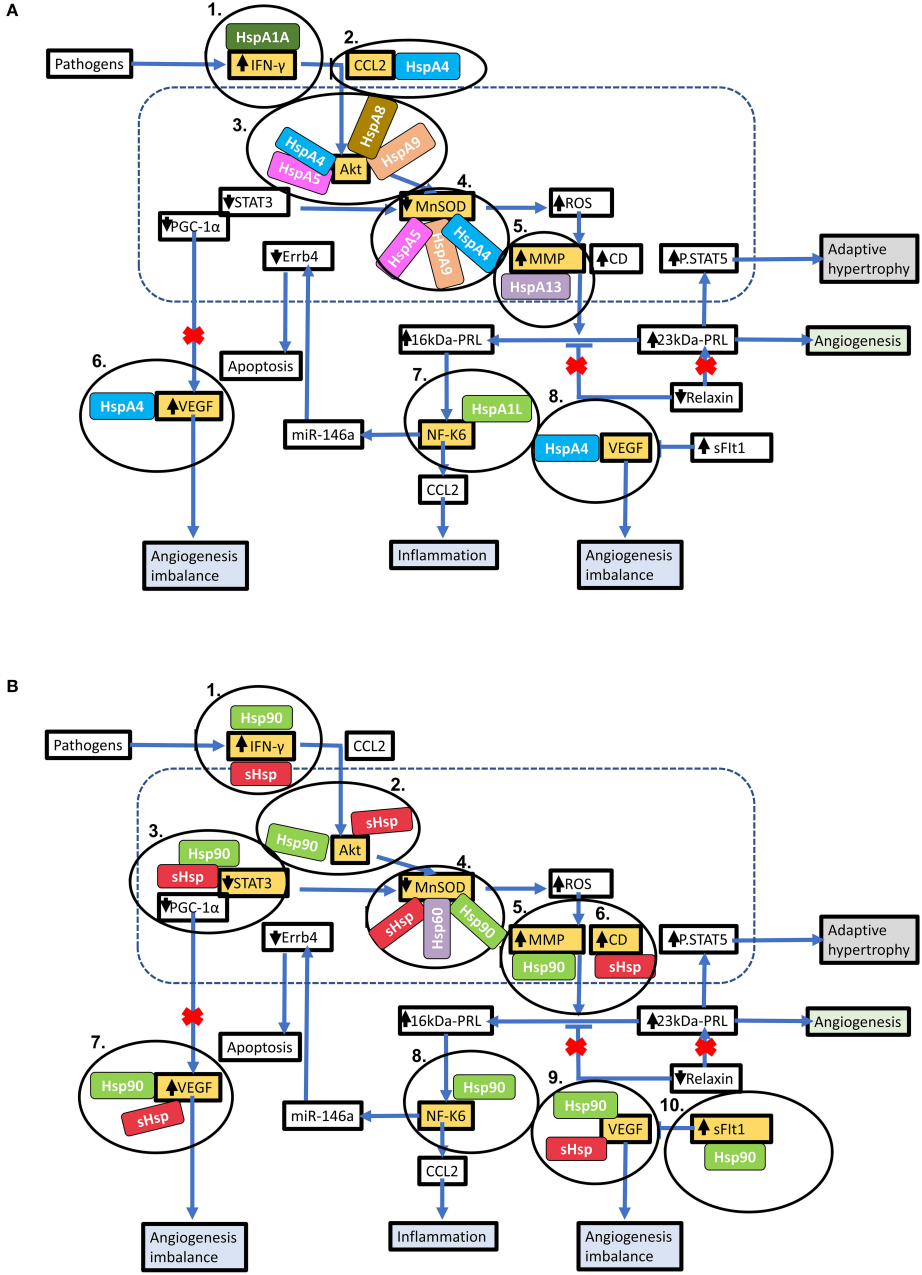
Key stages in the PPCM pathophysiology pathway which are modulated by Hsps. Hsps interact with several proteins involved in the PPCM pathophysiological pathway. **(A)** Several Hsp70 isoforms interact with various proteins involved in PPCM pathogenesis. **(B)** The stages within the PPCM pathophysiology pathway where Hsp90 and sHsps interact with PPCM proteins are shown. Targeting these stages may be crucial in novel PPCM interventions.

Hsps have previously been suggested as druggable candidates in human disease models (136). Several Hsps, such as Hsp70 and Hsp90 members, show great potential as drug targets in several cancers. For instance, the proteasome inhibitor, Bortezomib, has been used in anticancer therapy where it exerts antitumor effects by upregulating Hsp60 and Hsp90 on the surface of cancer cells (137). Attention is currently being drawn toward Hsp-directed therapies as candidates for novel cardiovascular disease therapies. A study recently demonstrated that blocking Hsp70 activity could be therapeutically beneficial in HF treatment (138). Given its ATP-dependent nature, Hsp70 is also amenable to inhibition using ATP-mimicking drugs. Interestingly, predictions from our STRING analysis revealed several stages which can potentially be modulated by Hsp70 (Figure 5A).

Hsp-centred drug modulation strategies may be centred around one of the following strategies; (i) boosting Hsp expression and (ii) inhibition of Hsp functionality. Each of these possible strategies is assessed in detail (Table 3). Inhibition would generally involve the development of small molecule inhibitors that target the functional domains of the Hsps thus disrupting their chaperone function. The direct inhibition of Hsps, resulting in the subsequent disruption of key protein-associations can be an efficacious way to modulate protein fate. Small molecule inhibitors such as polymyxin B and colistin sulphate possess great potential in this regard, as they have been successfully used to inhibit the activity Hsp70 *in vitro* (139). These two compounds are directed toward the nucleotide-binding domain, thus lowering the basal ATPase activity of the Hsp70. Designing domain specific inhibitory compounds may prove useful in Hsp70-targeted PPCM therapy. Since Hsp70 functions in co-operation with several other proteins involved in the pathophysiology of PPCM, selective targeting of cardiac Hsp70 may become a decisive step in inhibiting PPCM pathology. An alternative strategy worth exploring in designing novel PPCM therapy involves targeting heat shock factors (e.g., HSF1) which are responsible for modulation of Hsp expression. Although the generally high sequence and structural conservation of Hsp70 may be a snag in targeted inhibition, specific signature motifs in individual Hsp70 family members may be targeted toward this. Particularly, we predicted the association of several PPCM pathophysiology proteins with HspA4 (Figure 5A). HspA4 belongs to the Hsp110 family of chaperones, which are a specialised subclass of Hsp70s functionally and structurally distinguished from the canonical Hsp70s (56). This makes HspA4 amenable to selective targeting by inhibitors since it is unique from the more conserved canonical Hsp70s.

**Table 3:** Hsp-centred drug modulation strategies in novel PPCM drug design.

**Drug molecule**	**Mechanism of action**	**Possible cardiac implication**
**(i) Hsp expression enhancing drugs**		
Geranylgeranyl-Acetone	Induces Hsp70 and HspB8 expression	Cardioprotective effects have been reported in cardiomyopathy modes
Simvastain	Induces HspB1, Hsp70 and Hsp90 expression	Improves cardiac function and symptoms in DCM patients
**(ii) Hsp functionality inhibitors**		
Polymixin B	Inhibits Hsp90 and Hsp70 (NBD) chaperone function	May reduce inflammatory effects of Hsps on cardiac tissue
EGCG	Inhibits Hsp70 and Hsp90 expression by inhibiting the promoter activity of the respective chaperones	May reduce inflammatory effects of Hsps on cardiac tissue
Colistin sulphate	Inhibits Hsp90 and Hsp70 (NBD) chaperone function	May reduce inflammatory effects of Hsps on cardiac tissue

**Figure 6:**
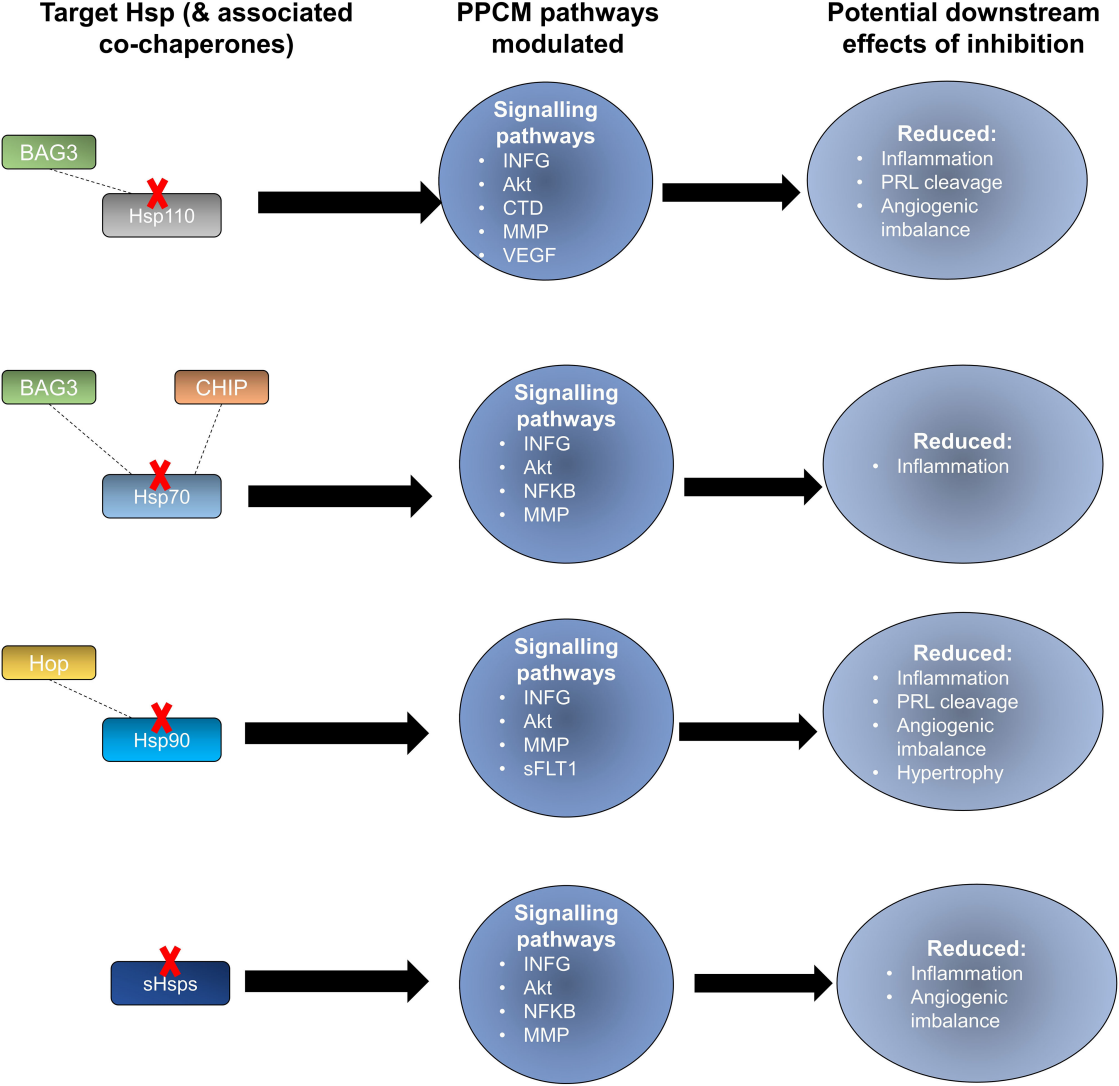
Potential effects of Hsp-directed inhibition in PPCM therapy. Targeting the Hsp types sHsp, Hsp110, Hsp90, and Hsp70 has the potential to disrupt key signalling pathways that are important in PPCM pathophysiology.

Prospects of targeting co-chaperones that are crucial for Hsp70 function also exist. The Hsp70 co-chaperone, CHIP, has been shown to play a crucial role in regulating intracellular protein signalling as evidenced by an increase in Akt phosphorylation which in turn leads to activation of the Akt signalling pathway leading to cardiac hypertrophy (63, 140). CHIP-directed therapies may potentially disrupt Hsp70 activity in turn causing detrimental downstream effects on Akt signalling within cardiomyocytes (Figure 6). Another Hsp70 co-chaperone, BAG3, may also be a crucial PPCM drug target. Disruption of the BAG3-sHsp-Hsp70 complex has previously been shown to be associated with DCM and non-inflammatory MFM (141–143). The specific roles of BAG3 in PPCM pathophysiology are however yet to be validated. BAG3 mutations have however been proposed to be associated with PPCM. Thus, enhancing BAG3 expression in PPCM patients may possibly offer cardioprotection by augmenting cardiomyocyte proteostasis. As such, it may therefore be generally hypothesised that these co-chaperones may act as decisive players in Hsp-modulated proteostasis within the cardiomyocyte and may thus act as PPCM drug targets.

Hsp90 also serves as an attractive candidate for novel PPCM drug interventions given that it is predicted to modulate the PPCM pathogenesis pathway (Figure 5B) via its interaction with AKT and NFKB1 (Figure 6). It is well-established that Hsp90 plays important functions in regulating client proteins into their active conformations. As such, Hsp90 possibly activates AKT triggering a cascade of events that leads to PPCM. Furthermore, Hsp90 is predicted to interact with NFKB which is associated with cardiac inflammation in PPCM. Hsp90 thus likely plays decisive roles in PPCM pathophysiology via AKT and NFKB signalling. Consequently, Hsp90-inhibiting directed therapies may prove beneficial in novel PPCM therapies. To date, several classes of Hsp90-targeting small molecule inhibitors have been proposed for disrupting Hsp90 chaperone function. The majority of these compete with ATP for binding onto the N-terminal domain (NTD) ultimately keeping the chaperone in an inhibitor-bound conformation that abrogates its function (144). Currently, the following natural and synthetic Hsp90-targeting drugs, such as ansamycin and derivatives of purine, resorcinol, benzamide, and tricyclic imidapyridines have been described in several disease models such as cancer [reviewed in (145)]. However, the potential of Hsp90-targeting drugs in PPCM is yet to be explored.

## Hsps As Candidate PPCM Biomarkers

**Figure 7:**
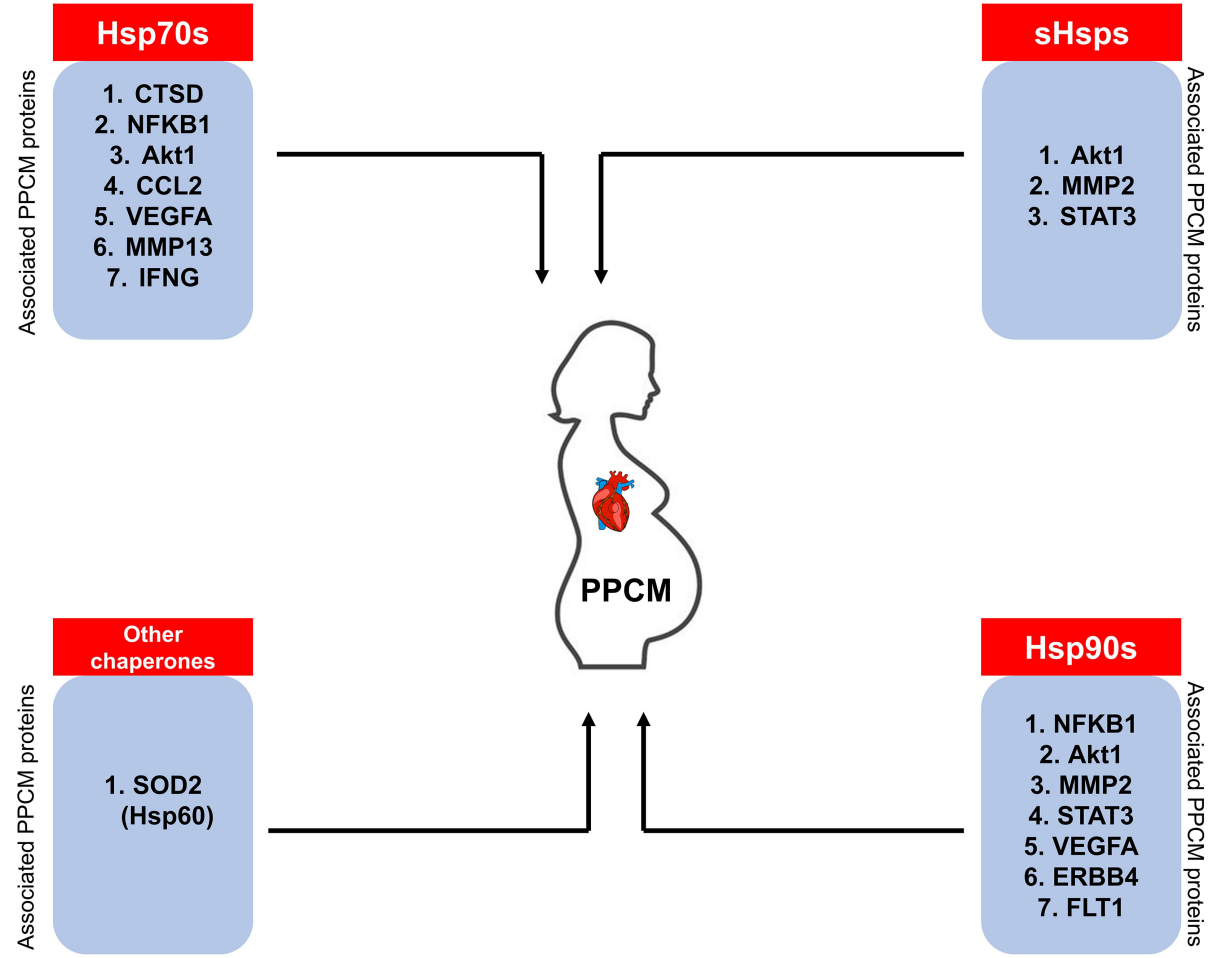
Hsps as potential PPCM biomarkers. The Hsps (sHsps, Hsp60, Hsp70, Hsp90) predicted to associate with PPCM pathogenesis proteins may be used as candidate biomarkers.

Despite the importance of early diagnosis for full cardiac recovery in PPCM patients, physicians are often faced with the difficulty of distinguishing between peripartum discomfort in healthy women and the pathological PPCM symptoms. PPCM diagnosis thus relies on a high index of suspicion. A thorough history of the onset of symptoms combined with a comprehensive echocardiography report confirming <45% LVEF are important diagnostic determinants. Currently, the only clinically confirmed PPCM biomarker is the brain natriuretic peptide NT (proBNP) which is however not specific for PPCM diagnosis (12, 146). Since Hsps are triggered by stress, we hypothesise that Hsp expression is upregulated to allow the chaperones to form functional partnerships with markers of inflammation in PPCM patients. It is important to monitor Hsp expression levels through the progression of PPCM as this presents a basis for the potential use of Hsps as biomarkers. Indeed, STRING analysis predicts the interaction of Hsp70, Hsp90, sHsps, and Hsp60 with several PPCM pathophysiology proteins (Figure 7). The prospects of Hsps as PPCM biomarkers either individually or in a panel with the already established proBNP marker is promising.

At present, some Hsps have been proposed as biomarkers in cardiovascular disease. A study by Giannessi and colleagues (37) demonstrated that circulating Hsp60 and HspA1A levels correlate with LV dysfunction severity. Interestingly, the same study also established that HspA1A protein levels are significantly correlated with BNP levels. Hsp70 and Hsp60 activation and inflammation markers, such as IL6 are also correlated with the extent of cardiac and microvascular dysfunction in patients with angiographically normal coronary arteries (37). Hsps have also been reported to mediate coronary endothelial dysfunction and produce microvascular damage in response to metabolic or infective insults (147). The potential of Hsp60 as a potential diagnostic or prognostic marker of heart disease has also been investigated. Veres et al. (148) noted the increased risk of heart disease when Hsp60 expression is upregulated. Also, elevated Hsp60 concentrations are positively associated with the severity of coronary arterial disease and ischemic heart disease in a dose-dependent fashion (149). Hsp70 levels were found to be significantly higher in HF and myocardial infarction, potentially implicating the protein's role as a CVD biomarker (150, 151). To date, not many proteomics-based studies have been conducted to assess the expression levels of stress proteins in PPCM. Nonetheless, the exacerbated stress associated with PPCM may trigger a unique chaperone response which may be studied toward biomarker design. Therefore, it may also be necessary to validate the role of other Hsp families such as the Hsp110 and Hsp40 members to determine their specific roles in PPCM. Recently, Hsp110 family members have been demonstrated to play important roles in cancers (152, 153). As such, the differential expression of Hsps in PPCM patients may lay a foundation toward novel biomarker identification.

Hsp70 may serve as an important diagnostic or prognostic biomarker of PPCM since Hsp70 expression levels are correlated with traditional injury markers such as AST, ALT, γGT and bilirubin in HF patients (154). Furthermore, a study by Baba et al. (155) predicted that worse outcomes correlate with increased Hsp70 levels after heart transplantation. Although similar studies have not yet been applied to PPCM at present, they are worth exploring. Given that there are 13 different Hsp70 isoforms which are subtly distinguished from each other by unique signature motifs, the Hsp70 family members are promising PPCM biomarkers. sHSPs have also been proposed as biomarkers of congestive HF. It has been demonstrated that Hsp20, Hsp27, and Hsp32 expression correlates to disease (156). The Hsp70 co-chaperone, BAG3 is also an attractive potential PPCM biomarker. Increased BAG3 levels were observed in sera of patients with end stage HF, purporting that BAG3 is released by cardiomyocytes as a stress response (157–159). As such, BAG3 may be a useful biomarker to monitor HF progression. The aggravated stress conditions associated with PPCM potentially trigger upregulated Hsp-expression to maintain proteostasis and alleviate the effects of cardiotoxicity. We therefore hypothesise an Hsp expression profile that is unique from other forms of cardiomyopathy toward the identification of novel PPCM biomarkers of diagnosis or prognosis. Indeed, while in HF several studies have focused on the potential role of Hsp60 and Hsp70, there is need to investigate the role of co-chaperones and other Hsps (e.g., Hsp40 and Hsp110) as PPCM biomarkers.

## Conclusion

The involvement of Hsps in protein quality control systems has been reported in several cardiovascular disorders such as ischaemic heart disease and HF. Given the exaggerated cardiac stress associated with pregnancy, Hsps likely play crucial roles in PPCM pathophysiology. Here, we predicted associations between proteins involved in PPCM pathogenesis and sHsp, Hsp70 as well as Hsp90. Furthermore, Hsps and their respective co-chaperones hold promise as novel candidate PPCM biomarkers. However, fundamental research is needed in this regard to experimentally validate the utility of Hsps as PPCM biomarkers. These associations could also pave the way for the development of novel Hsp-targeted therapeutic PPCM drug interventions.

## Author Contributions

GC, TS, and SK wrote the draft, the original idea was conceived by GC and KS. Critical feedback was provided by TZ, AS, NN, and KS. All authors contributed to the article and approved the submitted version.

## Conflict of Interest

The authors declare that the research was conducted in the absence of any commercial or financial relationships that could be construed as a potential conflict of interest.
